# Do health literacy, physical health and past rehabilitation utilization explain educational differences in the subjective need for medical rehabilitation? Results of the lidA cohort study

**DOI:** 10.1186/s12889-024-19086-5

**Published:** 2024-06-18

**Authors:** Jean-Baptist du Prel, Max Rohrbacher, Chloé Charlotte Schröder, Jürgen Breckenkamp

**Affiliations:** 1 Department of Occupational Health Science, University of Wuppertal, Wuppertal, Germany; 2 Department of Epidemiology & International Public Health, School of Public Health, Bielefeld University, Bielefeld, Germany

**Keywords:** Education, Subjective need for rehabilitation, Health literacy, Subjective health, Past rehabilitation utilization, Causal mediation, Behavioral model

## Abstract

**Background:**

Medical rehabilitation can be helpful for maintaining workers’ health and work ability. Its contribution to longer working lives is of high economic relevance in aging populations. In Germany, individuals must apply for rehabilitative measures themselves. Therefore, the subjective need for rehabilitation (SNR) is a prerequisite for rehabilitation access. A low education level is associated with poor health, lower health literacy and more frequent utilization of health services. In the present study, we investigated whether lower educational levels are also associated with a greater SNR and whether health literacy, past rehabilitation utilization and physical health play a mediating role in this path in older employees.

**Methods:**

3,130 socially insured older employees (born in 1959 or 1965) who participated in the German prospective lidA (leben in der Arbeit) cohort-study in 2011, 2014 and 2018 were included. A causal mediation analysis with an inverse odds weighting approach was performed with the SNR as the dependent variable; educational level as the independent variable; and health, health literacy and past rehabilitation utilization as the mediating variables. Sociodemographic variables were adjusted for.

**Results:**

The SNR was significantly greater in subjects with a low education level, poor physical health, inadequate health literacy and those who had utilized rehabilitation in the past. For health literacy, past rehabilitation utilization and physical health, a significant partial mediating effect on the SNR was found for employees with low compared to those with high education levels. However, the combined mediating effect of all the mediators was lower than the sum of their individual effects. Among those with medium or high education levels, none of the variables constituted a significant mediator.

**Conclusions:**

The path between a low education level and a high SNR is mediated by inadequate health literacy, past rehabilitation utilization and poor physical health; these factors do not act independently of each other. Promoting health education may lower the SNR by improving physical health and health literacy. While improving physical health is beneficial for individuals, improved health literacy can be economically advantageous for the health system by reducing inappropriate expectations of rehabilitation benefits and subsequent applications for rehabilitation.

**Supplementary Information:**

The online version contains supplementary material available at 10.1186/s12889-024-19086-5.

## Background

In most European countries, the population is aging due to increased life expectancy and low birth rates [1]. To safeguard social security systems in Germany, working lives are being extended, and early exit from the workforce is becoming more difficult [2]. It is estimated that more than 75% of workers over the age of 55 years have at least one chronic health condition that requires management [3]. Chronic health conditions or poor health in general have an impact on the ability to work. Medical rehabilitative measures are one approach to maintaining or restoring older employees’ ability to work and to avoid early exit from the labour force [4].

**Fig. 1:**
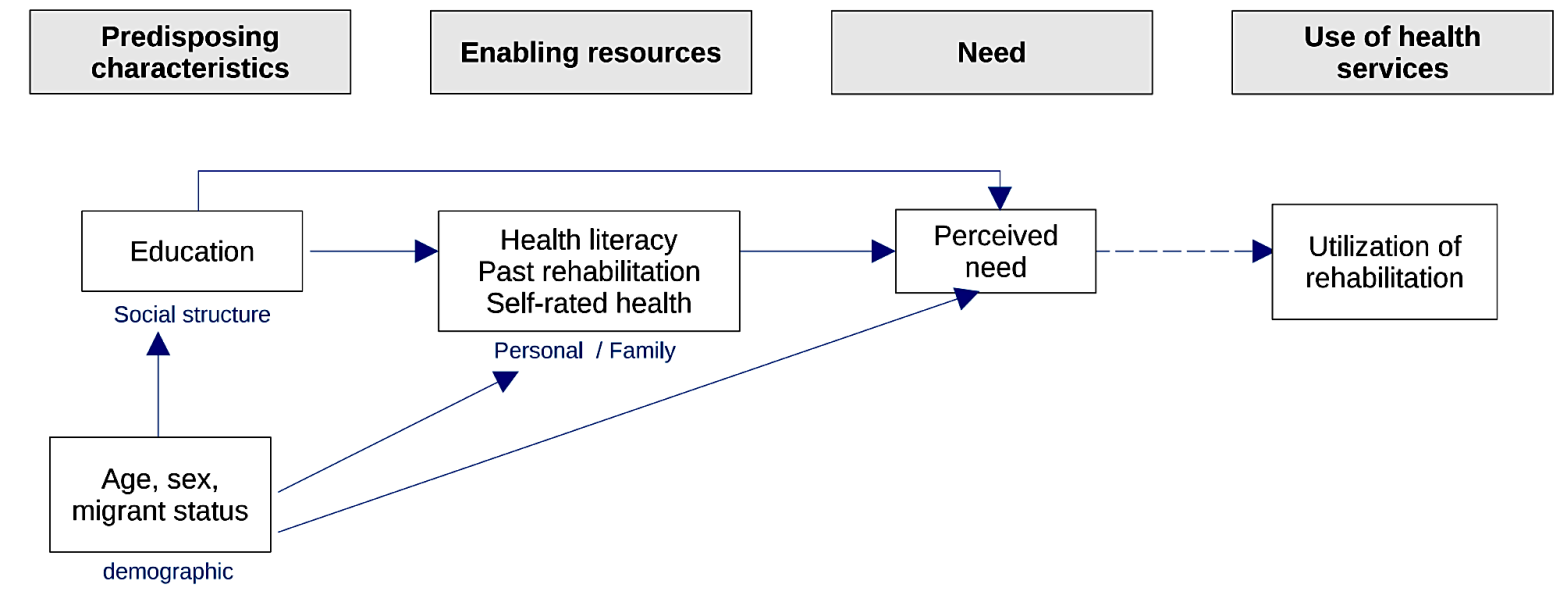
Theoretical model according to Anderson’s behavioral model (1995)

This study is motivated by Anderson’s behavioral model [5]. According to this model, the utilization of health services such as medical rehabilitation measures is preceded by various factors, which also represent the core area of Anderson’s model. These factors include predisposing characteristics, enabling resources and, as a consequence of these factors, the subjective need for healthcare services (Fig. 1). Our study is interested in this core area of Anderson’s model. Thereby the focus of our study is on individual factors that contribute to the subjective need for medical rehabilitation (SNR).

In an earlier analysis of German data on the utilization of healthcare services using Andersen’s model [5], need factors (e.g., multimorbidity, self-perception of health, injuries, pain) play the strongest role in utilization [6, 7]. In our model, we therefore assumed that the SNR would predict subsequent rehabilitation utilization to a certain degree and act as a proxy for it.

Regarding the predisposing characteristics, the focus of our investigation was on educational differences in SNR. There are only a few investigations about social inequalities in medical rehabilitation care [8, 9]. Health care provision in general can be assessed using the dimensions of access, utilization and quality. In the case of medical rehabilitation, earlier investigations have shown no or only weak associations between the utilization of rehabilitation services and social status [8]. A recent study by Fach et al. [9] revealed no significant differences in the chance of application when adjusting for health status and current employment situation. The existing results concerning rehabilitation utilization deviate from the social inequality observed in other health care domains. Therefore, it was interesting to investigate the association between educational status and SNR as a proxy for rehabilitation utilisation. To avoid confounding by other predisposing characteristics (age, sex, migration background) it was necessary to adjust for the influence of these factors in the relationship between education and the SNR.

With regard to individual enabling resources, our model emphasizes health literacy (HL). The assumption is that HL has an influence on the SNR and that it varies according to the level of education. Therefore, HL plays a mediating role in our model. Furthermore, according to Anderson’s model [5], subjective health cannot only be a target variable but also influence the relationship between predisposing factors and subjective needs. Moreover, it can be assumed that subjective health influences the SNR depending on the level of education. Therefore, we included subjective health in our model as a second mediator in the relationship between education and the SNR. Finally, as a further enabling resource, we assumed that rehabilitation utilization in the past could also change the SNR depending on the respondent’s level of education. Therefore, this parameter was a third mediator in our analysis model.

### The SNR does not always equal the actual need

In Germany, a person requiring a rehabilitative measure must apply for this purpose. The SNR is therefore a prerequisite for access to rehabilitation services. The SNR may depend on several factors, including the level of education. However, differences in the SNR may in turn depend on other (SNR-related) factors associated with individuals’ educational level, such as health literacy, physical health, and experiences gained during previous participation in rehabilitation.

The actual need for rehabilitation is then determined by the treating physician and by statutory pension insurance, statutory health insurance, statutory accident insurance or another provider of rehabilitation costs on an individual basis [10]. Access to rehabilitation is thus possible only after the need has been professionally established. Approximately one-third of annual applications are not approved by pension insurance providers, predominantly for medical reasons. The rate of application to approval indicates that SNR does not always meet an actual need [11].

### Possible contribution of HL

In Europe, as well as in Germany, nearly every second person older than 14 years has low health literacy (HL) [12]. Low HL is significantly more prevalent in individuals with low education levels [13]. According to the HLS-GER 2 study, 65.6% of the participants had low health competency in managing illness, which was much greater than that of medium-educated (45.4%) or higher-educated (37.1%) adults in Germany [14]. Moreover, approximately three-quarters of the lower educated were not able to assess the pros and cons of different treatment options. Given the inherent disadvantages for individuals and the health care system with low health competencies, improving HL through health education across the life course is a desirable goal [15]. Poorer HL is associated with higher costs in health care and poorer health outcomes [16]. In Germany, participation in medical rehabilitation depends heavily on HL. Patients with higher HL can better assess their real need of professional health support [17]. Differences in HL might be an explanation for differences in subjective and actual need for rehabilitation mentioned above. Given the association of HL with educational level, we hypothesize that HL mediates the relationship between educational level and SNR.

### Possible contribution of physical health

Furthermore, findings suggest that functional HL may serve as a pathway by which socioeconomic status (SES) affects health status, especially in lower SES groups [18]. The association between the level of education and individual self-assessed health, morbidity, and mortality has been demonstrated by numerous studies internationally and in Germany [19–25]. In general, people with a low education level, low occupational status, and/or low income are more frequently ill, are more likely to use health services, and have poorer treatment outcomes. Disease-related functional limitations are more common, and health-related quality of life is lower. Therefore, education level as other social status indicators, is considered as a main determinant of health. Health, in turn, is associated with the use of health services such as rehabilitation. This is possibly due to a health-dependent increase or decrease in the subjective need for treatment. The probability of health impairments increases with age, so the SNR will most likely be increased in older employees. To our knowledge, studies investigating the relationship between physical health and the SNR in older employees are rare. In a German cohort study of older employees with back pain, different pain and health problems due to greater pain intensity, more severe pain-related limitations, greater impairment due to comorbidity, and poor self-perceived current state of health were identified as predictors of the wish for rehabilitation [26]. Therefore, health is hypothesized to be a second mediating variable in the path between the exposure (educational status) and the outcome (SNR).

Exploring pathways that contribute to the explanation of educational differences in rehabilitation utilization can be helpful to lower social differences in the health of older employees, and to ensure efficient utilisation of health services.

Against this background, we investigated whether educational status is associated with SNR among older employees in Germany and to what extent HL, past medical rehabilitation and physical health contributed as potential mediators to the variation of this association.

## Methods

The present study investigated the mediating effects of HL, past medical rehabilitation utilization and physical health in the association between education and SNR. For the analysis, data from the lidA (leben in der Arbeit) study were used. The lidA study is a prospective cohort study on work, age, health, and labour participation, representative for older workers from the German Baby Boomer generation from the 1959 and 1965 birth cohorts with respect to age, sex, nationality, education, and occupation [27, 28]. In the first study wave (t_0_, 2011), the primary response rate, calculated as the ratio of completed interviews (*n* = 6,637) to the operational sample (*n* = 24,322) according to the definition RR5 of the American Association for Public Opinion Research, was 27.3% [28, 29]. Of the 6,637 completed interviews, 6,585 were valid. These respondents were eligible for the follow-up waves, which took place in 2014 (t_1_, *n* = 4,244) and 2018 (t_2_, *n* = 3,586). A more detailed description of the study and sampling procedures can be found elsewhere [27, 28, 30, 31]. The present analysis included persons participating in all three study waves (*n* = 3,232), excluding respondents without valid information on the included covariates (*n* = 102). Full data were available from 3,130 subjects.

**Fig. 2:**
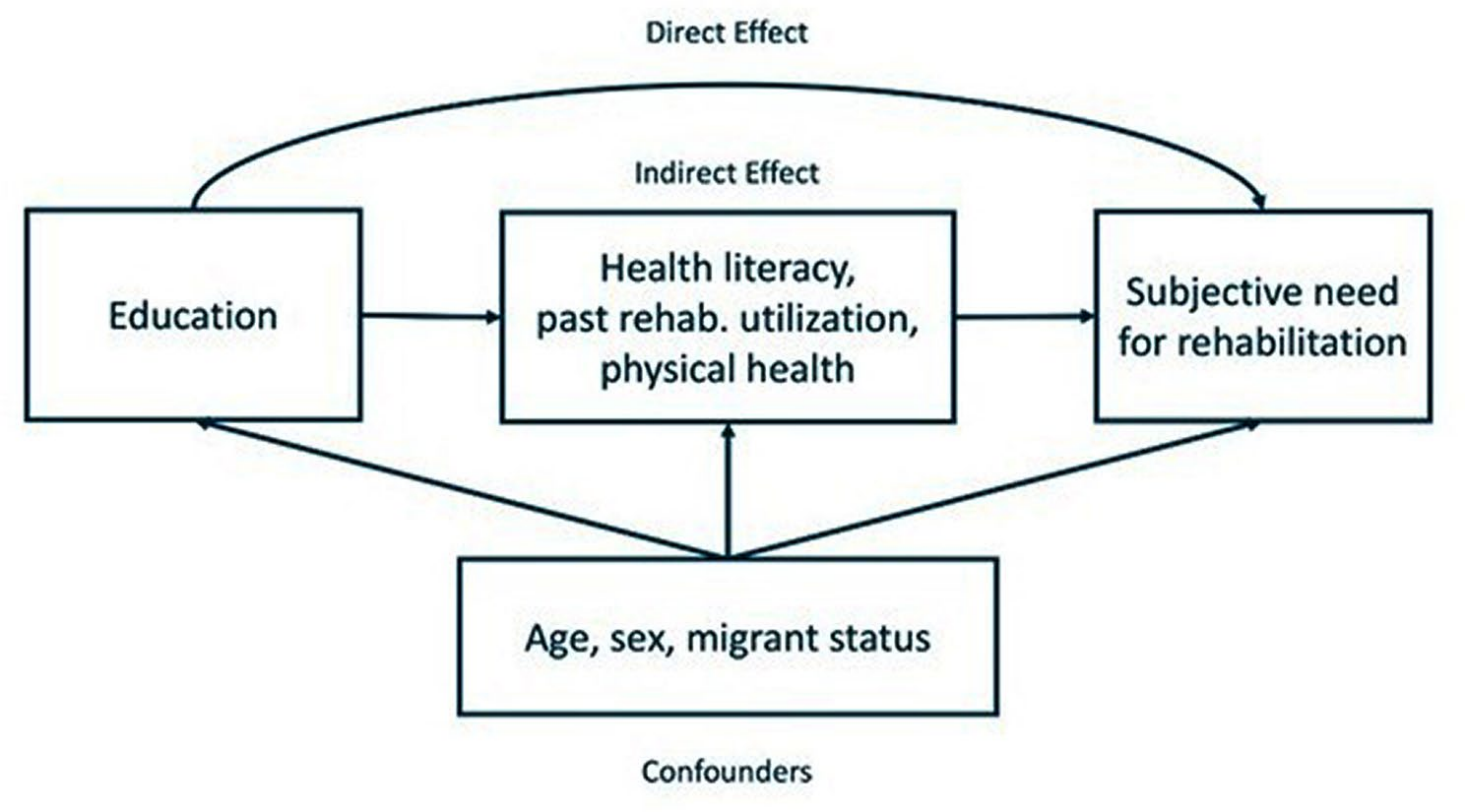
Analysis model with assumed causal relationships

Figure 2 shows the assumed causal relationship for our analysis model. With the description of the variables in the following paragraphs, the time of their measurement is indicated in parentheses. The arrows in the model in Fig. 2 describe both direct and indirect effects (mediated through HL, past rehabilitation utilization and physical health) of education on the SNR as well as the confounding influences on the exposure-mediator- and mediator-outcome associations.

### Subjective need for rehabilitation (t_2_)

The outcome of this analysis is the SNR. Participants were asked “Would you wish to participate in a rehabilitation programme (regardless of whether you have already had rehabilitation)?” The answer was restricted to “yes” or “no”. This item was assessed in study wave three (t_2_).

### Educational status (t_0_)

Educational status was considered the exposure. A score [32], which combines the level of schooling and vocational training, is used to determine the educational status attainment. The results are classified into three categories: high (tertiary education), medium (upper secondary vocational education and postsecondary nontertiary education) and low (primary, lower secondary and upper secondary general education). This item was assessed at wave one (t_0_).

### Health literacy (t_2_)

Health literacy was measured using the seven questions on the “coping with illness” dimension of the Health Literacy Questionnaire (HLQ) 16 [33]. Given the limited survey time, coping with illness was chosen, because it is the most important dimension in the context of our research question. This part of the HQL-instrument asks for the ability to find information about therapies for one’s own illness, whom to address professional medical aid, to understand the doctor’s or pharmacist’s therapeutic advice, to make one’s own health decisions on the basis of the doctor’s advice, to ask for a second medical opinion and to follow the doctor’s advice. The responses “very easy” and “fairly easy” were combined into “easy”, and “fairly difficult” and “very difficult” were combined into “difficult”. Each question answered with “easy” was scored with one point. Then, three categories were set up nearest to the categorization of the complete HLQ-instrument with 16 items defined by Röthlin et al. [33]: inadequate (0 to 3 points), problematic (4 to 5 points), and sufficient HL (6 to 7 points). This item was assessed in wave three (t_2_).

### Physical Health (t_2_)

Physical health was assessed with the Short Form Health Survey (SF-12). Based on the items, a physical component score (PCS_Score) was created as described by Nübling et al. [34]. The PCS_score (0-100) was subsequently divided into tertiles. The lowest tertile (PCS_Score < 43.86) was defined as the cut-off point for separating employees in poor physical health from those in good physical health. This item was assessed in wave three (t_2_).

### Past rehabilitation utilization (t_0_ – t_2_)

To assess past rehabilitation utilization (yes/no), participants were asked at each study wave whether they had previously participated in a rehabilitation measure. The answers reflect participation in the period between 2008 and 2018.

### Confounders

Age (born in 1959 or 1965), sex (female/male) and migrant status (no migrant background/1st generation migrants with German citizenship/1st generation migrants with foreign citizenship/2nd generation migrants) were considered confounders of the exposure-mediator and mediator-outcome associations. Persons who were born abroad and who subsequently immigrated were defined as having a 1st generation migrant background. Persons of the 2nd generation were born in Germany but had at least one parent who was born abroad [35].

### Statistical analysis

First, we conducted a descriptive analysis to display the sample characteristics and the proportion of participants with SNR. A chi-square test was used to test the associations between the covariates and the outcome (Table 1). P values were derived from Cramer’s V test.

**Table 1:** Sample characteristics by subjective need for rehabilitation (*n* = 3,130)

		Subjective need for rehabilitation		p value^a^
		No	Yes	
		*n* = 1,595	*n* = 1,535	
Education level	Low	293 (44.7%)	363 (55.3%)	< 0.001
	Medium	887 (49.9%)	891 (50.1%)	
	High	415 (59.6%)	281 (40.4%)	
Year of birth	1965	878 (51.8%)	817 (48.2%)	0.31
	1959	717 (50.0%)	718 (50.0%)	
Sex	Male	760 (54.8%)	626 (45.2%)	< 0.001
	Female	835 (47.9%)	909 (52.1%)	
Migrant status	None	1,373 (51.5%)	1,294 (48.5%)	0.013
	1st Gen German	68 (44.2%)	86 (55.8%)	
	1st Gen foreign	46 (63.9%)	26 (36.1%)	
	2nd Gen	108 (45.6%)	129 (54.4%)	
Health literacy	Sufficient	1,167 (55.5%)	934 (44.5%)	< 0.001
	Problematic	315 (43.9%)	402 (56.1%)	
	Inadequate	113 (36.2%)	199 (63.8%)	
Past rehab utilization (2008–2018)	No	1,212 (60.2%)	800 (39.8%)	< 0.001
	Yes	383 (34.3%)	735 (65.7%)	
Physical health	Good	1,229 (59.2%)	846 (40.8%)	< 0.001
	Poor	366 (34.7%)	689 (65.3%)	

Row percentages displayed; ^a^p values obtained from chi-square tests

To quantify the multiple mediating effects of HL, past medical rehabilitation and physical health in the association between education and SNR, an inverse odds weighting (IOW) approach [36] was used. This counterfactual-based approach to mediation has advanced during recent decades and has several advantages over traditional product / difference of coefficients approaches. The IOW approach allows for the decomposition of the total effect into direct and indirect effects in mediation analysis with binary outcomes and regardless of the presence or absence of an exposure-mediator interaction, which may be of particular importance when investigating a social status indicator such as education. Using the IOW, we can furthermore accommodate multiple mediators of any measurement scale simultaneously [37]. Detailed descriptions of the IOW approach of causal mediation including practical implications and examples are given by Nguyen et al. [37] and VanderWeele [38].

Using the counterfactual-based IOW approach, the total effect (TE) of the exposure (education) on the outcome (SNR) was decomposed into a natural direct effect (NDE) and a natural indirect effect (NIE). The NDE describes the effect of changing the exposure (e.g., from high to low education) but fixing the mediator. In this way, the indirect pathway through the mediator(s) is deactivated [37–40]. The NIE describes the effect of changing the mediator to the value it would naturally take had the exposure changed (e.g., from high to low education), while the exposure is actually fixed (direct pathway deactivation) [37–40].

In line with Nguyen et al. [37], the mediation was conducted in six consecutive steps. First, in an exposure model, education level was regressed on the mediator(s) and confounders using multinomial regression. To compute the inverse odds weight (IOW), the inverse of the predicted log odds for each observation in the exposed group was taken from this first model. This step included two postestimation predictions, one for the low educated and one for the medium educated. In the second step, the IOW was assigned to the exposed group (firstly low education level, then medium education level), and a weight equal to 1 was assigned to the reference group. Third, in an outcome model regressing the SNR on education level and confounders, the TE was estimated using a generalized linear model from the Poisson family and log link function. Poisson regression was preferred over logistic regression because, for nonrare outcomes (> 10%), the odds ratio is noncollapsible, leading to downward biased indirect effects [38, 41]. Fourth, the NDE was estimated using the same model but specifying the IOW. Afterwards, the NIE was obtained by subtracting the NDE from the TE. TE, NDE, NIE and their 95% confidence intervals were subsequently computed by bootstrapping with 1,000 replications. All steps were carried out for each mediator under investigation separately and combined, adding the mediators sequentially in a temporally plausible order. Importantly, to be able to interpret the obtained NDE and NIE causally, several assumptions about confounding had to be made. We assumed that there was no unmeasured confounding of the (i) exposure–outcome relationship, (ii) mediator–outcome relationship or (iii) exposure–mediator relationship; furthermore, we assumed (iv) the absence of a mediator–outcome confounder, which itself is affected by the exposure [42]. To determine the extent of effect mediation, the proportion mediated (PM) was calculated using the equation for ratio measures by VanderWeele [42], here for the relative risks (RR):

$$Proportion \,mediated \,\left(PM\right)=\frac{{RR}^{NDE}\times ({RR}^{NIE}-1)}{({RR}^{NDE}\times {RR}^{NIE}-1)}$$

The significance level was set at *p* < 0.05. Respondents with missing values for the included covariates were initially excluded from the analysis sample. Missing values for a single variable were max. 2.4% (HL). All analyses were conducted with Stata V15.1 (College Station, TX: StataCorp LLC).

### Sensitivity analysis

To check whether selection bias may have influenced the results, we repeated the analysis using a non-response weight. The weight accounts for selective dropout by inverse probability weighting for migrant status and education level. With this method, the data are standardized on the population of the lidA baseline in 2011. The weight is calculated for each subgroup by percentage in wave 1/percentage in wave 3. For example, for the group of non-EMB with high educational level 16.44%/18.03%=0.9116. The weighting factors for all other subgroups can be found in the supplementary table S1. In this case, the exposed group (first low education level, then medium education level) was assigned the IOW multiplied by the non-response weight, and the reference group was assigned the non-response weight only.

## Results

Complete data from 3,130 study participants were available for analysis. The dataset consists of 56% women and 44% men. 54% of the participants were born in 1965 (aged 46 years at t_0_ and 53 years at t_2_), and 46% were born in 1959 (aged 52 years at t_0_ and 59 years at t_2_).

Table 1 presents the characteristics of the study participants with reference to the SNR. The SNR was greater for study participants who had a low education level or were female. Additionally, inadequate HL, previous rehabilitation and poor physical health are associated with the SNR. Migration status was also significantly associated with the SNR, while age (year of birth) was only weakly associated with the SNR. Further descriptive analyses by educational status (Table 2) showed that the prevalence of poor physical health varied widely and significantly between educational groups, while only smaller but significant differences in the prevalence of inadequate health literacy were found to the disadvantage of lower educational groups.

**Table 2:** Sample characteristics of the study population by educational status (*n* = 3,130)

		Education			*p* value^a^
		Low	Medium	High	
		*n* = 656	*n* = 1,778	*n* = 696	
Year of birth	1965	317 (48.3%)	997 (56.1%)	381 (54.7%)	0.003
	1959	339 (51.7%)	781 (43.9%)	315 (45.3%)	
Sex	Male	345 (52.6%)	677 (38.1%)	364 (52.3%)	< 0.001
	Female	311 (47.4%)	1,101 (61.9%)	332 (47.7%)	
Migrant status	None	533 (81.3%)	1,553 (87.3%)	581 (83.5%)	< 0.001
	1st Gen German	34 (5.2%)	79 (4.4%)	41 (5.9%)	
	1st Gen foreign	28 (4.3%)	21 (1.2%)	23 (3.3%)	
	2nd Gen	61 (9.3%)	125 (7.0%)	51 (7.3%)	
Subjective need for rehab.	No	293 (44.7%)	887 (49.9%)	415 (59.6%)	< 0.001
	Yes	363 (55.3%)	891 (50.1%)	281 (40.4%)	
Health literacy	Sufficient	417 (63.6%)	1,186 (66.7%)	498 (71.6%)	0.005
	Problematic	156 (23.8%)	412 (23.2%)	149 (21.4%)	
	Inadequate	83 (12.7%)	180 (10.1%)	49 (7.0%)	
Past rehabilitation utilization (2008–2018)	No	386 (58.8%)	1,129 (63.5%)	497 (71.4%)	< 0.001
	Yes	270 (41.2%)	649 (36.5%)	199 (28.6%)	
Physical health	Good	345 (52.6%)	1,183 (66.5%)	547 (78.6%)	< 0.001
	Poor	311 (47.4%)	595 (33.5%)	149 (21.4%)	

Column percentages displayed; ^a^p values obtained from Chi-square tests

In Table 3, the decomposition of the effect of education level on the subjective need to participate in a rehabilitation measure into a TE, NDE and NIE is displayed. In addition to HL, we assumed that past rehabilitation utilization and poor physical health were relevant mediators. When comparing low- versus high-educated persons, HL accounted for 14% of the effect of education on the SNR (Table 3, analysis 1). Thus, if persons with a low education level had the same level of HL as did those with a high education level, irrespective of other potential mediators, the effect of education level on the SNR would be reduced from 1.37 (95%-KI 1.22–1.52) to 1.32 (95% CI 1.17–1.46). Past rehabilitation utilization explained 21% (Table 3, analysis 2), and poor physical health explained 31% (Table 3, analysis 3) of the effect of education on the SNR when the mediators were investigated separately. Next, we analysed effect mediation by adding the mediators sequentially into the mediation analysis. Adding past rehabilitation utilization to the model containing health literacy only (Table 3, analysis 4), 24% of the effect of education on the SNR could be explained (RR^NIE^ 1.07, 95% CI 1.03–1.11). Additionally, adding physical health explained 15% (hence, 39%) of the educational differences in SNR (RR^NIE^ 1.12, 95% CI 1.06–1.17) (Table 3, analysis 5).

**Table 3:** Causal mediation analysis of the association between education and the subjective need for rehabilitation* (*n* = 3,130)

	Low vs. high education level			Medium vs. high education level		
	RR	95% CI^a^	PM^b^ %	RR	95% CI^a^	PM^b^ %
TE of education	1.37	1.22–1.52		1.21	1.09–1.34	
**Analysis 1: health literacy**						
NIE	1.04	1.01–1.07	**14**	1.01	0.99–1.03	6
NDE	1.32	1.17–1.46		1.20	1.08–1.32	
**Analysis 2: past rehab. utilization**						
NIE	1.06	1.02–1.09	**21**	1.02	1.00–1.04	11
NDE	1.29	1.15–1.44		1.19	1.07–1.31	
**Analysis 3: physical health**						
NIE	1.09	1.05–1.14	**31**	1.01	0.99–1.03	5
NDE	1.25	1.11–1.40		1.21	1.08–1.33	
**Analysis 4: health literacy & past rehab. utilization**						
NIE	1.07	1.03–1.11	**24**	1.02	1.00–1.05	11
NDE	1.28	1.13–1.42		1.19	1.07–1.31	
**Analysis 5: health literacy & past rehab. utilization & physical health**						
NIE	1.12	1.06–1.17	**39**	1.02	0.99–1.05	11
NDE	1.23	1.09–1.37		1.19	1.07–1.31	

*Decomposition of the effect of education on the subjective need to participate in a rehabilitation measure into a total effect (TE), natural direct effect (NDE) and natural indirect effect (NIE) using health literacy, physical health, and past rehabilitation utilization as mediators. The proportion mediated was marked in bold if the respective NIE was significant

All analyses adjusted for age, sex and migrant status

^a^obtained from bootstrapping (1 000 reps)

^b^Proportion mediated = RR_NDE_*(RR_NIE_-1)/(RR_NDE_*RR_NIE_-1)

*Abr.* CI = confidence interval; NDE = natural direct effect; NIE = natural indirect effect; RR = relative risk; TE = total effect

A comparison of persons with medium versus high education levels showed that, in this case, none of the putative mediators significantly contributed to the effect of education level on the SNR when investigated separately or in combination.

### Sensitivity analysis

The results from the sensitivity analysis show only slight differences compared to the findings in Table 3, indicating that attrition had a very small influence on the effect estimates. Both the NIEs and NDEs were robust. According to the sensitivity analysis, HL mediated 14%, HL and past rehabilitation 24%, and all three mediators combined, including physical health, mediated 36% of the effect of low vs. high education level on the SNR (see supplementary Table S2). Similar to our initial analysis, there was no significant NIE through health literacy when comparing persons with medium versus high education level.

## Discussion

In accordance with previous research [33], we found that among older workers in Germany, functional HL acts as a mediator in the pathway between educational level and the SNR. Additionally, past rehabilitation utilization and physical health were identified as mediators in this pathway in our analysis. However, all three mediating effects were observed for older employees with a low education level but not for those with a medium education level in comparison to highly educated employees. Moreover, our analysis revealed that the three mediators may not act independently of each other in employees with low versus high education levels. The effect of all the mediators together was lower than the sum of their individual effects, indicating that the effect of one mediator might already be partly explained by the other.

Insufficient health literacy is a global problem. In 2004, the U.S. Institute of Medicine stated that 90 million Americans had difficulty understanding complex texts, with corresponding consequences for the use of information on health and the health care system [43]. However, basic literacy and numeracy skills are not sufficient to understand the complex processes related to individual health, from taking medication to applying for rehabilitation. Therefore, literalism falls short, and the approach has been extended to the broader concept of health literacy. A definition by Sørensen et al. [44] summarizing the essence of 17 definitions found in their review is “Health literacy is linked to literacy and entails people’s knowledge, motivation and competences to access, understand, appraise, and apply health information in order to make judgments and take decisions in everyday life concerning healthcare, disease prevention and health promotion to maintain or improve quality of life during the life course.” In our investigation, these competences are measured by the ‘coping with illness’ dimension of the HQL 16 [33] and are assumed to have an influence on the SNR.

In 2016, approximately 54% of the population in Germany reported having problems dealing with health-related information. Moreover, health literacy is unequally distributed: a higher proportion of people with a low education level have inadequate health literacy [14, 45, 46]. In accordance with these findings, we have found significant differences in health literacy to the disadvantage of older employees with low educational level.

When investigating HL and the SNR, one must keep in mind that there is always the risk of under- or overestimating needs. An underestimation of the need for rehabilitation services may occur when there is insufficient information about suitable measures [47]. On the other hand, overestimating the need implies that the SNR does not necessarily mean that rehabilitation will be helpful for people with a given health condition [47]. The observation that the percentage with SNR was much greater in older employees with insufficient HL than in those with sufficient HL (Table 1) may indicate an overestimation of the SNR in our investigation. Given that lower SES is associated with low HL [14, 45, 46], the greater SNR in older employees with low education levels might be partly explained by lower HL, probably overestimating the beneficial effects of rehabilitation on health. This could also be one of the reasons why one-third of rehabilitation applications are rejected mainly for medical reasons in Germany [11].

Furthermore, poor physical health contributes to educational differences in SNR. First, we observed a social gradient regarding physical health, measured by the 12-item Short Form Health Survey (SF-12), to the disadvantage of older employees with low educational level. This finding is consistent with numerous other studies about health inequality [19–25]. Then, in our investigation poor physical health was also significantly associated with a greater SNR (Table 1), or more specifically, with the wish to participate in a medical rehabilitation measure. As health status and its implicit limitations in daily life are the most important aspects of application behaviour and the approval of rehabilitation measures, this seems to be plausible [48]. The association between health and SNR is also in agreement with the findings of previous investigations on backpain [26]. In sum, the mediating effects of low HL and poor physical health on the association between low education and high SNR in our analysis seem to be plausible in light of the existing knowledge about the associations between the mediating variables and the independent or dependent variables in our model.

One plausible explanation for the partial mediation by past utilization of rehabilitation in this association might be that those with a greater need for rehabilitation in the past will also have a greater need, as well as the wish for rehabilitation, in the future. All associations in our model are in the expected directions. The observation in our analysis that the common mediating effect of HL, past rehabilitation utilization and physical health is weaker than the sum of their singular natural indirect effects might be explained by the fact that their effects act in the same direction and that one mediation is affected by the other [49]. Insufficient HL and poor health are associated with lower education and lead to a greater SNR. Insufficient HL is also associated with rehabilitation utilization and poor health, as found in earlier investigations [17, 50]. Assuming poor health, as well as HL to be stable over time to some extent, we hypothesize the following: Poor health could have been associated with rehabilitation utilization in the past and may be associated with a greater need and a wish for rehabilitation in the future. This may also explain why the combined mediating effect of low health literacy, poor physical health and past utilization of rehabilitation was lower than the sum of their individual effects because all of these factors at least partly address a greater need for rehabilitation because of poor health. Past utilization of rehabilitation was included in the analyses because it is known from the literature that health literacy and participation in rehabilitation are closely related [17] and, moreover, because participation in rehabilitation has an effect on the SNR.

Therefore, the natural indirect effect of poor physical health and past rehabilitation utilization on the association between education level and the SNR might be partly explained by the path through HL. If this is the case, the promotion of HL could be of notable importance in lowering the need for rehabilitation in older employees with low education level: On the one hand, better HL could improve physical health, and on the other hand, overestimating the SNR through inadequate health literacy could be avoided. Both could contribute to a lower SNR. According to our analysis, a considerable part of the association between a low education level and a high SNR could be eliminated by improving health literacy. A recent study on strengthening health literacy at the workplace (Geko-A) found that it is possible to significantly lower the percentage of employees with inadequate health literacy by an interactive learning toolbox [51]. Integrating knowledge about medical rehabilitation in those interactive learning units could also be helpful to improve health literacy, knowledge about rehabilitation and the health of older employees. Moreover, the workplace would be the right setting for these interventions to reach high-risk groups, such as the lower educated older workers.

While a special focus of this investigation is on the mediating effect of health literacy, regarding the proportion mediated by health in relation to HL, it is important to note that the improvement of physical health by other (primary preventive) measures could also contribute to a reduction in the SNR in less educated employees compared to older employees with higher education levels.

## Strength & limitations

One strength of the lidA study is its representativeness of socially insured employees born in 1959 or 1965 working in Germany [27]. Furthermore, the study provides longitudinal data for a high number of study subjects across three study waves. Another strength is the great variety of study characteristics included in the lidA study, which were measured with validated instruments such as the SF12 for physical health. This approach was useful for adjusting for several potential confounders in our analysis. As described in the methods section, several assumptions about confounding factors had to be made. The first three assumptions (i, ii, iii) require temporal ordering of the analysis variables; i.e., the exposure and the mediators precede the outcome (i, ii), and the mediators succeed the exposure (iii) [37, 42]. Hence, another advantage of the study inherent to its longitudinal design is that these variables could be measured at different points in time. The exposure education was measured temporally prior to the mediators and outcome, together with the pre-exposure confounding variables, age, sex, and migrant status. The mediators were measured at follow-up at the same time as the outcome. Physical health was assumed to have an influence on the SNR without a time lag. HL data were collected in the 3rd study wave (t_2_) for the first time, and this was considered to be a relatively stable characteristic and hence likely temporally preceding the other mediators. Mediation analysis using an inverse odds weighting (IOW) approach [36] is adequate for studying joint indirect effects through multiple mediators. This approach overcomes some of the disadvantages of other approaches, such as the difference-in-coefficients approach. For example, the IOW approach is applicable for models with a non-linear link function, regardless of a possible exposure-mediator interaction on the outcome [39]. Furthermore, mediators could be investigated separately and simultaneously, which is advantageous since the sum of the separate mediation effects is rarely an appropriate approximation of their combined effect, given their dependence conditional on the exposure [49]. The findings from our simultaneous analysis supported our assumption of a possible sequential order of the mediators, i.e., health literacy and past rehabilitation utilization affect the outcome through physical health. A disadvantage of this approach is, however, that this assumption cannot be tested explicitly; rather, it can be derived only from the order in which mediators are investigated and the logic of the causal ordering between these mediators, which was mentioned above for our model. As noted in an earlier study using the IOW method [52], only in the model containing all mediators (Table 3, analysis 5) were the obtained NDE and NIE robust to the unmeasured common causes of the investigated mediators. Therefore, especially the full model may be causally interpreted. Finally, concerning assumption four (iv), the risk of confounding of the mediator-outcome association affected by the exposure was minimized, given that none of the observed confounders could be exposure-induced. However, given the time lag between the baseline and follow-up measurements, the existence of such a confounder cannot be conclusively ruled out.

In addition to these merits, our study has several limitations. Only socially insured employees born in 1959 and 1965 were included. While the majority of employees in Germany are socially insured [27], nothing about sworn civil servants, self-employed individuals or employees of other age groups can be said. The primary response rate of 27.3% was in line with the declining willingness to participate in population-based surveys over the last several decades in Germany [53, 54]. However, the sample was in 16 different socio-demographic variables highly representative to the data of the ‘Integrated Employment Biographies’ (IEB), where the study sample was drawn from and which includes all employees subject to social security in Germany [28, 30]. A source of bias in this study could be loss to follow-up. Despite the sensitivity analysis with a dataset with inverse probability weighting for education level and migration status, attrition bias for other variables cannot be ruled out.

Finally, the transferability of our results to other countries globally, may be limited by substantial differences in the coverage, financing, organization and practice of medical rehabilitation [55]. One essential difference of the German health system in comparison to other European countries, is the access path to medical rehabilitation. As mentioned initially a person, requiring a rehabilitation measure in Germany must apply for it. In most other European countries, a physician either in the clinic or at the workplace decides about the person’s need and carries out the access to medical rehabilitation [56]. In some countries, like Finland, the duration of absence from work is reason to ascertain whether there is an individual need for rehabilitation [57]. Regarding these differences in access, the subjective need for rehabilitation may be a better proxy for the utilization of rehabilitation in Germany than in other countries.

## Conclusion

According to our findings, the greater SNR in older employees with a low education level might be reduced by improving their HL and physical health. An advantage of improving HL in older employees with a low education level would be that it simultaneously has a positive influence on physical health. Interactive learning units at the workplace might improve HL and other measures to improve physical health can also be helpful in lowering the SNR in older employees with low education levels. Future research should verify our model in countries with different cultural and economic background including health systems with different access to rehabilitation.

### Electronic supplementary material

Below is the link to the electronic supplementary material.

Supplementary Material 1

Supplementary Material 2

## Data Availability

The datasets for the first two waves of the lidA study are available as a scientific use file at the research data centre of the German Federal Employment Agency at the Institute of Employment Research. Further information can be found here: https://fdz.iab.de/en/our-data-products/archived-data/lida/ [58]. The scientific use file for study wave 3 is still in preparation and will be available for researchers at the Research Data Centre of the German Statutory Pension Insurance in the near future. Additional information regarding the study as well as data documentation (data reports and methods reports) are also available [27, 28, 30, 31].

## Abbreviations

CI: Confidence interval
HL: Health literacy
HLQ: Health literacy questionnaire
IOW: Inverse odds weighting
IEB: ‘Integrated Employment Biographies’
lidA: ‘Leben in der Arbeit’
NDE: Natural direct effect
NIE: Natural indirect effect
PB: Percentage mediated
PCS_Score: Physical component score
RR: Relative risk
SNR: Subjective need for rehabilitation
SES: Socioeconomic status
t_0_: Study wave 1 (2011, baseline)
t_1_: Study wave 2 (2014)
t_2_: Study wave 3 (2018)
TE: Total effect
