# Supplementary material for: Do health literacy, physical health and past rehabilitation utilization explain educational differences in the subjective need for medical rehabilitation? Results of the lidA cohort study
Source: BMC Public Health. 2024 Jun 18;24:1622. doi: 10.1186/s12889-024-19086-5 (PMC11186266; doi:10.1186/s12889-024-19086-5)
Supplement: Supplementary file 1 — Supplementary Material 1 [file 12889_2024_19086_MOESM1_ESM.docx]

**Supplementary Table S1:** Weighting factors for inverse probability weighting

|  | Non-EMB | German  G1 EMB | Foreign  G1 EMB | G2 EMB |
| --- | --- | --- | --- | --- |
| Education level |  |  |  |  |
| High | 0.9116 | 1.1969 | 0.9817 | 0.8796 |
| Medium | 0.9260 | 1.4299 | 1.5472 | 0.9059 |
| Low | 1.1346 | 1.8080 | 1.8991 | 1.0136 |
| Non-EMB: employees without migrant background  German G1 EMB: 1^st^ generation migrants with German citizenship  Foreign G1 EMB: 1^st^ generation migrants with foreign citizenship  G2 EMB: 2^nd^ generation migrants | | | | |
