# Supplementary material for: Do health literacy, physical health and past rehabilitation utilization explain educational differences in the subjective need for medical rehabilitation? Results of the lidA cohort study
Source: BMC Public Health. 2024 Jun 18;24:1622. doi: 10.1186/s12889-024-19086-5 (PMC11186266; doi:10.1186/s12889-024-19086-5)
Supplement: Supplementary file 2 — Supplementary Material 2 [file 12889_2024_19086_MOESM2_ESM.docx]

**Supplementary Table S2:** Weighted causal mediation analysis of the association between education and the subjective need for rehabilitation (n= 3 130)*

|  | low vs. high education level | | |  | medium vs. high education level | | |
| --- | --- | --- | --- | --- | --- | --- | --- |
|  | RR | 95% CI^a^ | PM^b^ % |  | RR | 95% CI^a^ | PM^b^ % |
| TE of education | 1.37 | 1.22-1.52 |  |  | 1.21 | 1.09-1.34 |  |
| Analysis 1: health literacy |  |  |  |  |  |  |  |
| NIE | 1.04 | 1.01-1.07 | **14** |  | 1.00 | 0.98-1.01 | 0 |
| NDE | 1.31 | 1.17-1.46 |  |  | 1.22 | 1.09-1.35 |  |
| Analysis 2: past rehab. Utilization |  |  |  |  |  |  |  |
| NIE | 1.06 | 1.02-1.09 | **21** |  | 1.01 | 0.98-1.04 | 11 |
| NDE | 1.30 | 1.15-1.44 |  |  | 1.20 | 1.07-1.33 |  |
| Analysis 3: physical health |  |  |  |  |  |  |  |
| NIE | 1.10 | 1.05-1.15 | **33** |  | 1.05 | 1.02-1.07 | **27** |
| NDE | 1.25 | 1.10-1.40 |  |  | 1.16 | 1.03-1.28 |  |
| Analysis 4: health literacy & past rehab. utilization |  |  |  |  |  |  |  |
| NIE | 1.07 | 1.03-1.11 | **24** |  | 1.02 | 0.99-1.05 | 11 |
| NDE | 1.28 | 1.13-1.42 |  |  | 1.19 | 1.06-1.31 |  |
| Analysis 5: health literacy & past rehab. Utilization & physical health |  |  |  |  |  |  |  |
| NIE | 1.12 | 1.06-1.17 | **39** |  | 1.06 | 1.03-1.10 | **33** |
| NDE | 1.23 | 1.08-1.37 |  |  | 1.14 | 1.02-1.27 |  |
| *Decomposition of the effect of education on the subjective need to participate in a rehabilitation measure into a total effect (TE), natural direct effect (NDE) and natural indirect effect (NIE) with additional non-response weight.  The proportion mediated was marked in bold if the respective NIE was significant.  All analyses adjusted for age. sex and migrant status  ^a^obtained from bootstrapping (1 000 reps)  ^b^Proportion mediated = RR_NDE_*(RR_NIE_-1)/(RR_NDE_*RR_NIE_-1)  Abr.: CI = confidence interval; NDE=natural direct effect; NIE=natural indirect effect; RR = relative risk; TE= total effect. | | | | | | | |
